# Identification of the Potential Biological Preservative Tetramycin A-Producing Strain and Enhancing Its Production

**DOI:** 10.3389/fmicb.2019.02925

**Published:** 2020-01-14

**Authors:** Yinglong He, Yu Ding, Qingping Wu, Moutong Chen, San’e Zhao, Jumei Zhang, Xianhu Wei, Youxiong Zhang, Jianling Bai, Shuping Mo

**Affiliations:** ^1^Guangzhou Institute of Chemistry, Chinese Academy of Sciences, Guangzhou, China; ^2^Guangdong Open Laboratory of Applied Microbiology, Guangdong Provincial Key Laboratory of Microbial Culture Collection and Application, State Key Laboratory of Applied Microbiology Southern China, Guangdong Institute of Microbiology, Guangdong Academy of Sciences, Guangzhou, China; ^3^Guangzhou Institute of Chemistry, University of Chinese Academy of Sciences, Beijing, China; ^4^Department of Food Science and Technology, Institute of Food Safety and Nutrition, Jinan University, Guangzhou, China

**Keywords:** bio-preservative, *Streptomyces albulus*, tetramycin A, antifungal activity, high-throughput screening, metabolic precursor

## Abstract

The aim of this study was to develop a potential microbial preservative to prevent the growth of fungi in food. The isolate ZC-G-5 showed strong antifungal activity against food spoilage fungi and *Streptomyces albulus* was identified on the basis of morphologic, culture, and 16S rDNA sequence analyses. The active metabolite was elucidated as tetramycin A (TMA) through spectroscopic techniques, including HR-ESI-MS, 1D-NMR, and 2D-NMR. An antifungal activity assay revealed that the minimum inhibitory concentration (MIC) and minimum fungicidal concentration (MFC) of TMA were 1.50–2.50 and 3.00–5.00 μg/ml, respectively. *In situ* antifungal activity analyses demonstrated that 90.0 μg/ml of TMA could inhibit the growth of fungi for over 14 days. In order to enhance TMA production, the high-yield mutant strain YB101 was screened, based on the isolate ZC-G-5, using a high-throughput screening method. The best metabolic precursor was selected during fermentation, when the concentration of glycerol was 8% (v/v) in Gauze’s broth medium to cultivate the mutant strain YB101; the concentration of TMA could be increased to 960.0 μg/ml, compared with the original isolate ZC-G-5, where the concentration of the TMA was only 225.0 μg/ml. Our study may contribute to the application of *S. albulus* and its active metabolite as a potential bio-preservative in the food industry.

## Introduction

Food spoilage, especially caused by fungi is undesirable since the remaining fungi can produce mycotoxins that induce illness on consumption (Gupta et al., 2011; Vanhoutte et al., 2016). Some mycotoxins have highly carcinogenic properties, such as aflatoxin, a genotoxic, immunotoxic, and hepatocarcinogenic secondary metabolite produced mainly by *Aspergillus* (Bhatnagar et al., 2006; Fountain et al., 2018). One of the methods controlling food spoilage is to use preservatives, such as chemical ones (Eklund, 2010). However, both the food industry and consumers are becoming more aware of the environmental and health concerns associated with the application of chemical preservatives (García et al., 2010). Therefore, there is now an increasing demand for more natural products with lower rates of chemical treatment. Alternatives, such as bio-preservation, have gained increasing attention in recent years (Gálvez et al., 2007; Lucera et al., 2012; Silva et al., 2018). Bio-preservation technologies use natural or controlled microbiota or antimicrobials to preserve food and extend shelf-life. Usually, beneficial microorganisms and their fermentation products are used in bio-preservation to render spoilage microorganisms in food inactive (Randazzo et al., 2009; Bai et al., 2016).

Among the actinomycetes, *Streptomyces* sp. is a particular favorite owing to its antimicrobial activity against serious pathogenic and food spoilage microorganisms (Hayakawa et al., 2004; Balasubramanian et al., 2017). Natamycin is first isolated from *Streptomyces natalensis* and belongs to a group of polyene macrolide antimycotics (Van Leeuwen et al., 2009). Owing to its broad spectrum, lower toxicity, and lack of development of resistance, the U.S. Food and Drug Administration (FDA) approved the use of natamycin as a food bio-preservative (Singh, 2018). ε-polylysine is a homopolymer of 25 to 35 L-lysine residues, it is also produced by *Streptomyces* sp. with a broad antimicrobial spectrum that includes gram-positive and gram-negative bacteria, and yeasts (Yamanaka et al., 2010). It was also recommended as food preservative by the FDA and classified as GRAS (generally regarded as safe) (Morten et al., 2014). In our previous studies, ε-polylysine was also purified from the *Streptomyces griseofuscus* GIM8 strain and produced through *in situ* fermentation by resin-based, which provided the basis for large-scale industrialized production (Liu, 2012).

Tetramycin that contains two components (tetramycin A and tetramycin B) is the secondary metabolites of *Streptomyces* sp. (Radics et al., 1982). Tetramycin exhibits significant antifungal activity and has been used to treat fungal diseases in plants in China (Gao et al., 2018). Tetramycin A (TMA) is a 26-member tetraene macrolide compound which is the most efficient antifungal ingredient of tetramycin (Dornberger et al., 1979). Traditionally, the low productions of the secondary metabolites have limited the application of the active metabolites. In order to improve the productions of active metabolites, a variety of strain improvement strategies have been developed. All of these approaches yield a population of mutants with a diverse range of properties. Therefore, it is imperative to develop a high-throughput screening method to evaluate a large number of mutants and screen high-yield strains (Jun et al., 2013; Shi et al., 2015). What is more, metabolic precursors have important regulatory effects on target products during the fermentation process. Bajaj studied the effect of addition of different amino acids and tricarboxylic acid cycle intermediates as metabolic precursors to enhance production of poly (γ-glutamic acid) from *Bacillus licheniformis*. Cheng improved Vitamin B12 fermentation process by adding rotenone to regulate the metabolism of *Pseudomonas denitrificans* (Sanchez and Demain, 2002; Bajaj and Singhal, 2009; Cheng et al., 2014). According to the biosynthetic pathway, small molecule compounds of polyhydroxy play important role in the biosynthesis of macrolides among the actinomycetes (Recio et al., 2006). However, to the best of our knowledge, little study is available on *Streptomyces albulus* for enhancing TMA production by high-throughput screening method or selecting the best suitable metabolic precursor during the fermentation.

With this study, we (1) isolated and identified TMA-producing strain with significant antifungal activity against food spoilage fungi, (2) purified the TMA and identified its molecular structure based on HRMS and a combination of one-dimensional NMR and two-dimensional shift-correlated NMR techniques, (3) determined the effectiveness of the TMA in controlling food spoilage microorganism *in situ*, (4) screened the high-yield mutant strain YB101 based on the original isolate ZC-G-5 through high-throughput screening method and selected the best suitable metabolic precursor during the fermentation.

## Materials and Methods

### Isolation and Identification of the Active Strain

The strains were isolated from soil samples collected from the Baiyun Mountain in Guangdong, China, according to a previous study with some modifications (Holmalahti et al., 2010; Chaudhary et al., 2013). The food spoilage fungus strains *Penicillium citrinum* (GIM 3.458), *Aspergillus flavus* (GIM 3.271), *Aspergillus niger* (GIM 3.412), *Penicillium funiculosum* (GIM 3.551), and *Mucor mucedo* (GIM 3.456) that inoculated on potato dextrose agar (PDA) media were obtained from the Guangdong Institute of Microbiology.

The morphological characteristic of the isolate was determined by Scanning Electron Microscopy (S-3000N, Hitachi, Japan). Cultural and physiological characterization of the isolate was carried out by the method of the *International Streptomyces Project* (Ningthoujam et al., 2009). For 16S rDNA identification, the DNA of the isolate was extracted according to the instructions of the bacterial DNA extraction kit (Dong Sheng Biotech, Guangzhou, China). The 16S rDNA gene was amplified using the universal bacterial primers 27f (5′-GTGCTGCAGAGAGTTTGATCCTGGCTCAG-3′) and 1492r (5′-CACGGATCCTACGGGTACCTTGTTACGACTT-3′). The PCR products were then sequenced (Invitrogen, Shanghai, China). After editing and verifying the sequences manually, the closest relatives of the most common sequences were identified using online BLAST software and the GenBank database of NCBI^1^. A neighbor joining phylogenetic tree was constructed using the program MEGA6.0 (Center for Evolutionary Functional Genomics, United States) (Gu et al., 2016).

### Purification and Determination the Structure of the Active Metabolite

Culturing of the strain ZC-G-5 was performed in Erlenmeyer flasks with Gauze’s broth medium, shaking culture at 28°C, 120 r/min, 7 days. The cell-free supernatant liquid was obtained from 20.0 L of the fermentation broth and centrifuged at 8,000 r/min for 10 min at 4°C.

The macroporous adsorption resin D101 was placed in a glass column (5 cm × 100 cm) to separate the active metabolite from the cell-free supernatant liquid with a flow rate at 3.0 ml/min. When adsorption was accomplished, the elution liquid with concentration gradients of 10, 30, 50, 70, and 90% alcohol mixed with water was collected respectively and concentrated in rotary evaporator under reduced pressure at 40°C to get different crude extracts. The active fraction was purified by Waters’ preparative HPLC with Waters’ C18 column (19 mm × 100 mm, 5 μm) at 30°C. The mobile phase was methanol and water in gradient mode as follows: 0–5 min, 50% methanol; 5–10 min, 60% methanol; 10–20 min, 70% methanol; 20–25 min, 90% methanol. The flow rate was maintained at 15.0 ml/min and UV detection at 305 nm. The peaks of the different fractions were collected respectively and activity fraction was selected by tracing antifungal active.

The chemical structure of the active metabolite was determined by high resolution mass spectroscopy (HRMS) and nuclear magnetic resonance (NMR) spectroscopy, using a combination of one-dimensional NMR (^1^H NMR and ^13^C NMR) and two-dimensional shift-correlated NMR techniques (CQSY, HSQC, and HMBC), for the complete ^1^H and ^13^C signal assignments. Before NMR, the antifungal substance was dissolved in *d*_6_-methanol. ^1^H NMR and ^13^C NMR spectra were measured at 500 and 125 MHz, respectively, with tetramethylsilane (TMS) as the internal standard (Zhao et al., 2010). The HR-ESI-MS equipped with an electro-spray ionization (ESI) source was applied with a positive ion source (ESI^+^).

### Antifungal Activity Assay

According to the CLSI (formerly NCCLS) references M38A, the MICs and MFCs of TMA was determined by the micro-dilution broth method, two different kinds of food preservatives, natamycin, and potassium sorbate (AR, Macklin Corporation) as the positive controls (Dylag et al., 2010). Fresh cultures with five kinds of food spoilage fungi were used to prepare the spore suspensions, which were adjusted to 2.0 × 10^4^–4.0 × 10^4^ cfu/ml. The 96-well microplates were incubated aerobically at 28°C, the results were measured after 72 h. MIC was defined as the lowest concentration that showed a visual turbidity of less than or equal to 80% inhibition compared with that produced by the control well. The lowest concentration of the antifungal substance that killed ≥99.9% of the initial inoculum was defined as the MFC end point (Pisano et al., 2016).

For *in situ* antifungal activity assay, three kinds of foods of about 100 g including bread, citrus fruits, and tomato sauce were sprayed the mixed spoilage fungi liquid with colony-forming unit (cfu) of 2.0 × 10^4^–4.0 × 10^4^ cfu/ml about 10.0 ml and then randomly separated into seven groups stored in stomacher bags, respectively(*n* = 3). The initial concentration of antifungal substance was serially diluted to 180.0, 90.0, 45.0, 22.5, and 11.25 μg/ml by sterile distilled water. Each concentration of active metabolite about 10.0 ml was also sprayed on the surface of the three kinds of food as pre-treatment samples. The positive control groups were sprayed 200.0 μg/ml of natamycin solution about 10.0 ml. Natamycin can effectively inhibit the growth of molds, yeasts, and other fungi in food and can effectively extend the preservation time of food. Due to the high sugar content in most of the food, it is easier to promote the growth of microorganisms, mainly molds. Therefore, natamycin as a bio-preservative is widely used in cheese, meat products, drinks, and other food preservation. In this study, the three kinds of food are all within the range of these preservatives, those foods used in our daily life and easy to breed mold because of high sugar and high water content. The blank control groups were sprayed sterile water about 10.0 ml. The stomacher bags with different food samples were stored at 28°C with relative humidity of 40–50%. Food spoilage was determined according to the presence of microorganism colonies on the surface of the food. *In situ* antifungal activity of TMA was evaluated macroscopically by analyzing the relationship between the different concentrations and the time taken for the food to spoil (Tian et al., 2015).

### Enhancing the Tetramycin A Production

Enhancing TMA production was achieved by means of high-throughput screening of the mutant strain with high TMA production and selecting the most suitable metabolic precursors during the fermentation process. Before high-throughput screening strategy, the type of multi-well microtiter plate (MTP) which is most suitable for cultivating mutant strains should be selected. The MTPs including 96-well, 48-well, 24-well, and 12-well MTP were used to cultivate the isolated strain, the control group was Erlenmeyer flask 150 ml. The volume of Gauze’s broth medium in each container was 0.3, 1.5, 3.0, 6.0, and 50.0 ml, respectively. After being cultivated at 28°C for 7 days, the MTP in which the average concentration of TMA was close to the control group, was selected to culture the mutant strains in the next step.

**FIGURE 1:**
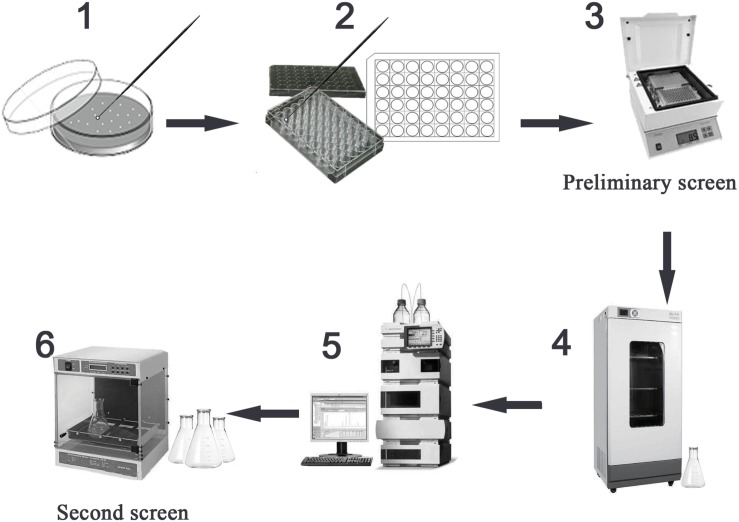
High-throughput screening the mutant strain with high TMA production. Preliminary screen included the steps 1–3 and second screen included the steps 4–6. The step 1 was to select the mutant strains with well growth, the mutant strains were obtained under UV repeated mutation five times, every time 2 min and cultivated on Gauze’s agar medium with 2% LiCl at 28°C for 3 days. The step 2 was to inoculate the mutant strains to the Gauze’s broth medium in 48-well MTP. The step 3 was to screen the potential high-yielding strains by microplate reader at 305 nm testing the cell-free supernatant liquids after cultivated at 28°C for 7 days. The step 4 was to cultivate the potential high-yielding strains. The step 5 was to determine the yield of TMA produced by high-yielding strains and exclude the interference of false positive strains by HPLC. The step 6 was to stabilize the yield of TMA produced by high-yielding strain through continuous passage culture.

In order to determine the feasibility of high-throughput screening strategy to detect the potential high-yielding strains, the concentrations 400.0, 200.0, 100.0, 50.0, and 25.0 μg/ml of TMA were successively added to the 96-well microtiter plate; the volume in each well was 200 μl. The relationship between the gradient concentrations of TMA and the OD value at 305 nm by microplate reader (Biotek, United States) was established. Therefore, during the process of high-throughput screening strategy, the preliminary screen was operated by a microplate reader to screen the potential high-yielding strains from the amounts of mutant strains, the mutant strains were obtained by UV repeated mutation five times, every time 2 min and cultivated on Gauze’s agar medium with 2% LiCl at 28°C for 3 days. The second screen was operated by HPLC to determine and exclude the interference of false positive strains. The flow chart of the strategy for the high-throughput screening of the mutant strain with high TMA production is shown in Figure 1.

Under the optimum conditions of fermentation, ethanol, glycol, propylene glycol, glycerol, and *n*-butanol were selected as the metabolic precursors to be added to the fermentation mediums, respectively. The control group used the fermentation medium without any metabolic precursors. The optimum conditions of fermentation were shake-flask cultivation, 150 ml of Gauze’s broth mediums was added into 500 ml of Erlenmeyer flask at inoculation of 15%, 160r/min, 28°C for 7 days. The initial volume ratios of the metabolic precursors were 5%. Based on the production of TMA, the best optimum metabolic precursor was selected after fermentation. At last, the best optimum metabolic precursor was selected as a single factor control during the fermentation, the best optimum concentration of the metabolic precursors was determined.

### Statistical Analysis

All assays were replicated three times for each treatment. Experimental data were analyzed using the statistical software Origin 8.0. Because of the limitations of the serial dilution technique, it was not possible to determine a cfu of less than 1 × log10 cfu/ml. The MIC and MFC values were calculated using the forecast function of Microsoft Excel (Microsoft Corporation, Redmond, Washington, United States). All values were expressed as means ± standard deviations (*n* = 3), and means were compared by Duncan’s multiple range test at 5% level of confidence, following one-way ANOVA test.

## Results

### Isolation and Identification of the Active Strain

A single strain, named as ZC-G-5, was selected by the agar block method for further antifungal activity analyses based on its zone of inhibition (mm) and preserved in Guangdong culture collection center (GDMCC), GDMCC NO.60301. Table 1 showed the zone of inhibition (mm) of the isolate against food spoilage fungi. Above these results, the isolated ZC-G-5 was selected for further study.

**TABLE 1:** Inhibition zone of the isolated strain ZC-G-5 against food spoilage fungi.

**Indicative strains**	***A. niger* (GIM 3.412)**	***A. flavus* (GIM3.271)**	***P. citrinum* (GIM 3.458)**
Diameter of inhibition zone (mm)^a^	16.51 ± 0.22	14.05 ± 0.36	22.10 ± 0.25

*^*a*^Numbers represent the means and standard deviations of the inhibition zones in mm obtained in three independent experiments.*

**FIGURE 2:**
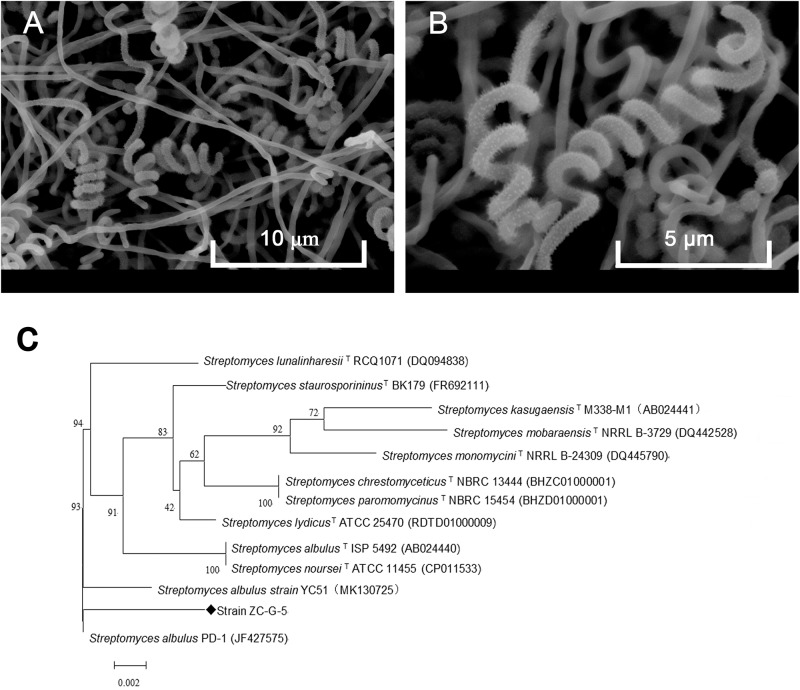
Identification of the isolate. Phenotype of the isolate ZC-G-5 growing on Gauze’s medium after 3 days at 28°C by scanning electron microscopy at 5,000× **(A)** and 10,000× **(B)**, respectively. Phylogenetic tree of the strain ZC-G-5 based on 16S rDNA sequences **(C)**.

The isolate grew well on most of the tested organic and synthetic media (Table 2). The colony had a hard surface with white aerial mycelia and gray spores, which had light purple and brown water-diffusible pigments present. The spore chains of the strain formed spirals, the spore was characterized by smooth surface, morphologically cylindrical, with a size of 0.5–0.6 × 0.8–1.0 μm (Figures 2A,B). To confirm the identity of the isolated strain, 16S rDNA sequence of the local isolate was compared with the sequences of 12 *Streptomyces* strains. The 16S rDNA sequence of strain ZC-G-5 showed high similarities to those of *S. albulus* PD-1 (97.87%), *Streptomyces lunalinharesii* RCQ 1071^T^ (96.86%), *Streptomyces lydicus* ATCC 25470^T^ (96.80%). The phylogenetic tree (Figure 2C) indicated that the strain was closely related to *S. albulus*. The 16s rDNA sequence has been submitted to the NCBI nucleotide data bases, the GenBank accession number for the nucleotide sequence is MN519495.

**TABLE 2:** Characteristics of the isolate ZC-G-5 and its product description^a^.

**Medium**	**Growth situation**	**Medium mycelia**	**Aerial mycelium**	**Soluble pigment**
Gause’s media	Well-grown	Hoar off-white	Gray	No
Soya peptone	Well-grown	Yellow	White	Faint yellow
ISP1	Well-grown	Yellow	White	Faint yellow
ISP2	Well-grown	Yellow	Gray	Yellow
ISP3	Well-grown	Creamy yellow	Gray	Faint yellow

*^*a*^ISP1, Peptone yeast extract agar; ISP2, Yeast extract agar; ISP3, Oatmeal agar.*

### Purification and Determination the Structure of the Active Metabolite

**FIGURE 3:**
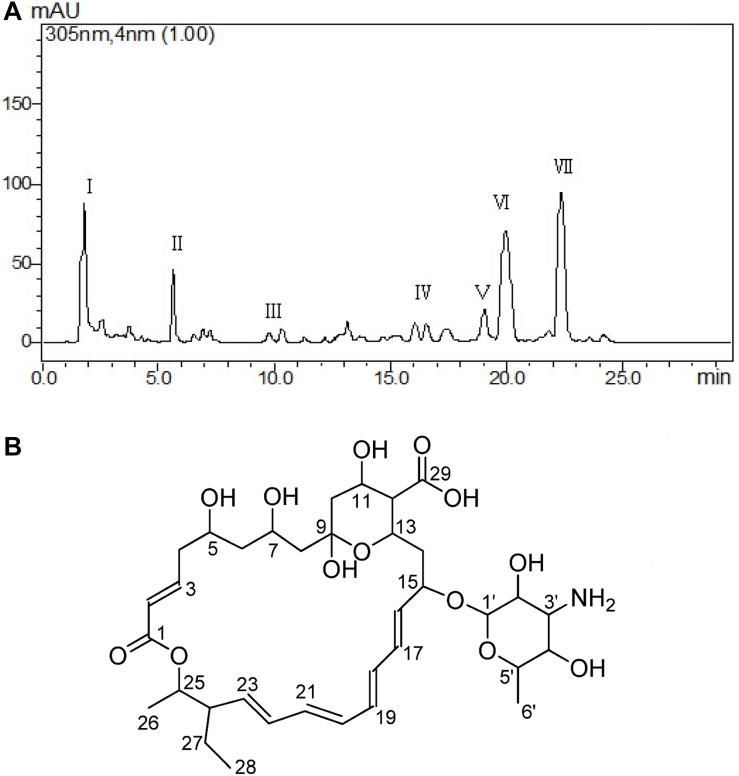
Purification and determination the structure of the active metabolite. Different fractions obtained by Waters’ preparative HPLC and fraction VII showed significant antifungal activity **(A)**. Structure of TMA according to NMR **(B)**.

The cell-free supernatant liquid was adsorbed by the macroporous adsorption resin D101; the fractions of crude extract with 50 and 70% alcohol elution solutions possessed strong antifungal activity. Those two fractions were combined about 8.5 g and purified at the next step. The different fractions were obtained by Waters’ preparative HPLC and termed as I–VII fractions (Figure 3A). In total, seven fractions were collected, and the yields of each fraction (I–VII) were about 13.50, 8.60, 2.30, 2.80, 3.20, 18.50, and 22.10 mg. The result of antifungal activity showed that fraction VII possessed the strongest antifungal activity. At last, the antifungal substance(about 22.10 mg)was purified from 20.0 L fermentation broth.

The antifungal substance was a white powder after freeze-drying. The UV spectrum of the antifungal substance in methanol showed maximum values at 291, 305, and 318 nm. HR-ESI-MS indicated molecular ion peaks at m/z 696.3611 [M + H]^+^ and 718.3426 [M + Na]^+^. The molecular formula of the antifungal substance was established as C_35_H_53_NO_13_. The chemical shifts of ^1^H and ^13^C (δ H and δ C) were calculated by the software MestReNova (Table 3). The spectral data (δ in ppm, J in Hz) of ^1^H-NMR at 500 MHz and ^13^C-NMR at 125 MHz were measured with CD_3_OD as the solvent. The antifungal substance was identified as tetramycin A (TMA) based on the above analysis and comparison with data from the literature (Dornberger et al., 1979; Radics et al., 1982). The picture (Figure 3B) showed the molecular structure of the TMA. NMR spectra of TMA(Supplementary Figure 1), including ^1^H-NMR spectrum, ^13^C -NMR spectrum, CQSY NMR spectrum, HSQC NMR spectrum, and HMBC NMR spectrum were determined.

**TABLE 3:** Nuclear magnetic resonance assignment of TMA^a^.

**Position**	**δH(mult, J in Hz)**	**δC**	**Position**	**δH(mult, J in Hz)**	**δC**
1	–	167.0	17	6.09(m, 1H)	136.4
2	5.78(d, 15.5, 1H)	125.4	18	6.39(m, 1H)	134.5
3	6.75(m, 1H)	147.5	19	6.25(m, 1H)	132.7
4	2.26(m, 2H)	42.9	20	6.38(m, 1H)	134.4
5	3.85(tt, 9.9, 2.3, 1H)	72.6	21	6.26(m, 1H)	133.3
6	1.49(m, 2H)	45.1	22	6.07(m, 1H)	137.9
7	4.43(m, 1H)	70.4	23	5.47(dd, 15.0, 9.7, 1H)	132.2
8	1.72(dd, 13.9, 11.2, 1H)	48.2	24	2.37(m, 1H)	49.6
	1.55(m, 1H)		25	4.97(m, 1H)	73.7
9	–	99.1	26	1.12(d, 6.6, 3H)	14.0
10	2.00(m, 1H)	45.2	27	1.38(m, 2H)	25.2
	1.25(m, 1H)		28	0.90(t, 7.5, 3H)	12.8
11	4.26(dt, 10.5, 4.7, 1H)	68.0	29	–	180.5
12	1.98(m, 1H)	62.0	1′	4.55(br, 1H)	98.9
13	4.40(br, 1H)	67.4	2′	3.99(br, 1H)	69.3
14	2.27(m, 1H)	39.4	3′	3.12(dd, 9.5, 2.5, 1H)	57.1
	1.57(m, 1H)		4′	3.34(m, 1H)	70.8
15	4.37(m, 1H)	79.7	5′	3.30(m, 1H)	74.5
16	6.14(m, 1H)	130.5	6′	1.26(d, 5.7, 3H)	17.8

*^*a*^Spectral data (δ in ppm, J in Hz) of 1H-NMR at 500 MHz and 13C-NMR at 125 MHz are measured.*

### Antifungal Activity Assay

**FIGURE 4:**
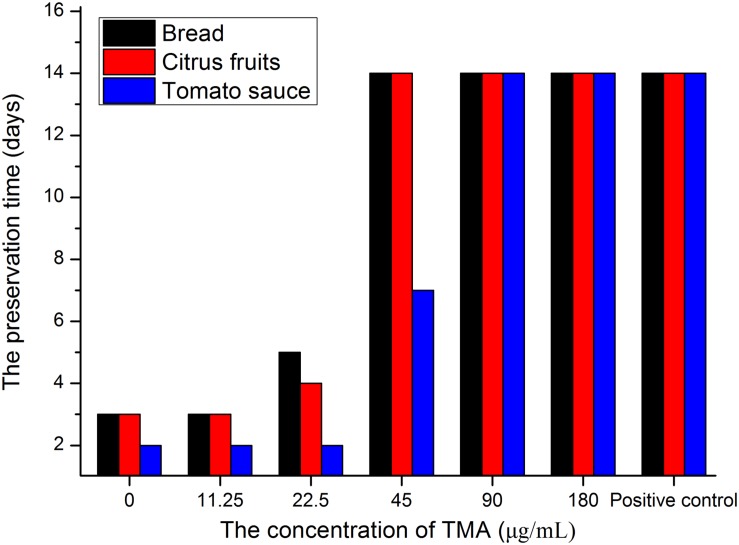
*In situ* antifungal activity assay of TMA. The blank control groups were sprayed sterile water about 10.0 ml, positive control groups were sprayed with 200.0 μg/mL of natamycin solution about 10.0 ml.

Table 4 summarized that TMA exhibited strong inhibition activity against five kinds of food spoilage fungi. The MIC and MFC of TMA against food spoilage fungi ranged from 1.50 to 2.50 μg/ml and 3.00 to 5.00 μg/ml, respectively. Natamycin is natural, highly efficient with low toxicity, and widely used as a bio-preservative in the food industry. Compared with the antifungal activity of natamycin, TMA possessed nearly the same activity against the tested organisms. *In situ* antifungal activity analyses showed that about 90.0 μg/ml of TMA could inhibit food spoilage fungi growing on the surface of bread, citrus fruits, and tomato sauce in the stomacher bags stored at 28°C with relative humidity of 40–50%, the preservation time was 14 days (Figure 4). However, when the concentration of TMA was under 45.0 μg/ml, the preservation time was less than 7 days. If there was no pretreatment of these foods, the preservation time was less than 3 days. Compared with 200.0 μg/ml of natamycin according to the application concentration of GB2760, the diluted cell-free fermentation supernatant with about 90.0 μg/ml of TMA was also enough to inhibit the growth of fungi on the surfaces of bread, citrus fruits, and tomato sauce for over 14 days.

**TABLE 4:** Comparison of TMA, natamycin, and potassium sorbate activities against food spoilage fungi^a^.

**Indicative food spoilage fungi**	**Food preservative**	**MIC (μg/mL)**	**MFC (μg/mL)**
*A. niger* (GIM 3.412)	TMA	2.25	4.50
	Natamycin	2.25	4.50
	Potassium sorbate	60.0	120.0
*A. flavus* (GIM3.271)	TMA	1.75	3.50
	Natamycin	2.25	4.50
	Potassium sorbate	77.50	151.0
*P. citrinum* (GIM 3.458)	TMA	1.50	3.00
	Natamycin	1.75	3.50
	Potassium sorbate	65.0	130.0
*P. funiculosum* (GIM3.551)	TMA	2.25	4.50
	Natamycin	2.00	4.00
	Potassium sorbate	72.5	145.0
*M. mucedo* (GIM3.456)	TMA	2.50	5.00
	Natamycin	2.25	4.50
	Potassium sorbate	72.5	145.0

*^*a*^MIC, minimum inhibitory concentration; MFC, minimum fungicidal concentration.*

### Enhancing the Active Metabolite Production

**FIGURE 5:**
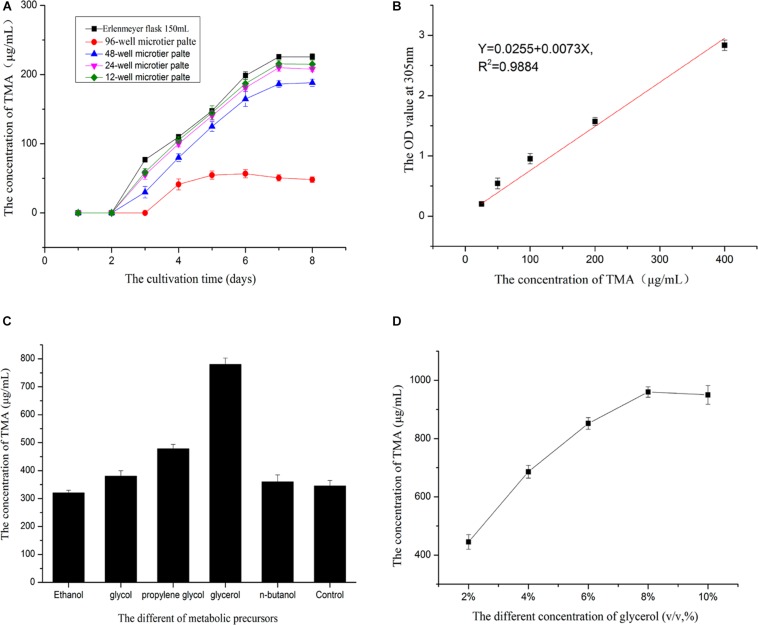
Enhancing the active metabolite production. Before high-throughput screening strategy, the MTP of the best suitable for cultivating mutant strains was be selected **(A)**. The relationship between the gradient concentrations of TMA and the OD value at 305 nm by microplate reader was established **(B)**. Selected the best optimum metabolic precursor **(C)**. The best optimum concentration of the metabolic precursor was determined **(D)**.

Cultivated the strain by MTP showed that 48-well microtiter plates was the best suitable for cultivating mutant strains(Figure 5A). 96-well microtiter plates could not replace the Erlenmeyer flask may be its volume too small to cultivated the strain. The relationship between the gradient concentrations of TMA and the OD value at 305nm was established(Figure 5B), liner curve equation: *Y* = 0.0255 + 0.0073*X*,*R*^2^ = 0.9884,*X* was the concentration of TMA,*Y* was the OD value of the solution at 305 nm. The linear curve showed the feasibility of high-throughput screening strategy to detect the potential high-yielding strains by the microplate reader. Therefore, the screening process was operated according to the flow chart of Figure 1; the mutant strain with high TMA production was screened. The concentration of the TMA was maintained at 385.0 μg/ml when the mutant strain YB101 continuous passage was cultivated three times after the preliminary screen and the second screen. Figure 5C showed that the concentration of the TMA was influenced by the different metabolic precursors. The small molecule compounds with polyhydroxy have the ability to promote the biosynthesis of TMA, but the addition of glycerol has an obvious effect on promoting metabolism. When the concentration of glycerol was 8% (v/v) in Gauze’s broth medium to cultivate the mutant strain, the concentration of the TMA could be increased to 960.0 μg/ml (Figure 5D), compared with the original strain ZC-G-5 that the concentration of the TMA was only 225.0 μg/ml.

## Discussion

In recent years, there has been an increasing demand for effective, safe, and natural products to control food spoilage without chemical residues (Al-Reza et al., 2010). As food bio-preservatives, natamycin and ε-polylysine isolated from *Streptomyces* have already been put into the market for many years (Sun et al., 2015). Actinomycete *Streptomyces* was well known for its potential to produce a large number of inhibitory metabolites and used especially in the food and pharmacology industries. With the progress of society and the development of technology, other natural products with potential bio-preservative produced by *Streptomyces* need to be researched in order to fulfill the rapid development of the food industry.

Although the antifungal substance was known, the isolate showed strong antifungal activity against food spoilage fungi as a major product providing an important biotechnological downstream advantage. There were no reports about *S. albulus* to produce TMA that enriched the diversity of active metabolites of *S. albulus* and TMA-producing strains. In this study, we reported that the antifungal substance TMA produced by *S. albulus* could act as a potential bio-preservative against food spoilage. However, any new bio-preservative must undergo rigorous toxicology tests and be approved by the FDA before it can be marketed, including natamycin and ε-polylysine. As a result, toxicology testing was needed before TMA could be used as a food preservative. Fortunately, natamycin as a bio-preservative was regarded as natural, highly efficient with low toxicity, and widely used in the food industry. In terms of molecular structure, TMA only has two additional methyl groups on polyene macrolides compared to natamycin. Similar molecular structure determines the same antifungal mechanism. What is more, antifungal activity assay showed that natamycin and TMA possessed almost the same antifungal activity. The next step, toxicology tests must be carried out in order to verify that TMA could represent an alternative to the use of natamycin as the bio-preservatives of food products.

Due to the satisfactory antifungal properties of TMA, enhancing the active metabolite production has an important prospect for its application. In this study, it was achieved through high-throughput screening of the mutant strain with high TMA production and selecting the best suitable metabolic presupposition for the optimization of cultivation situation. Before the high-throughput screening strategy, 48-well microtiter plates were the most suitable for cultivating mutant strains. The breeding of high-yielding strains can be achieved by preliminary screening with microplate reader and second screening with HPLC. The best metabolic precursor was selected during fermentation; when the concentration of glycerol was 8% (v/v) in Gauze’s broth medium to cultivate the mutant strain YB101, the concentration of the TMA could be increased to 960.0 μg/ml, compared with the original isolate ZC-G-5 when the concentration of the TMA was only 225.0 μg/ml. In the next step, we will improve the yield of TMA by molecular means, gene knockout, and other methods on the basis of the high-yield strain.

## Conclusion

In summary, the potential biological preservative Tetramycin A-producing strain was identified as *S. albulus* on the basis of morphologic, culture, and 16S rDNA sequencing analyses. The active metabolite was elucidated as TMA by means of spectroscopic techniques, including HR-ESI-MS, 1D-NMR, and 2D-NMR. Antifungal activity assay revealed that the MIC and the MFC of TMA against food spoilage fungi were 1.50–2.50 and 3.00–5.00 μg/ml, respectively. *In situ* antifungal activity analyses demonstrated that 90.0 μg/ml of TMA could inhibit the growth of fungi in three kinds of food for over 14 days. In order to enhance TMA production, high-yield mutant strain YB101 was screened based on the isolate ZC-G-5 through high-throughput screening method. The best metabolic precursor was selected during the fermentation, when the concentration of glycerol was 8% (v/v) in Gauze’s broth medium to cultivate the mutant strain YB101; the concentration of the TMA could be increased to 960.0 μg/ml, compared with the original isolate ZC-G-5 when the concentration of the TMA was only 225.0 μg/ml. The results of the present study indicated that TMA produced by *S. albulus* had a promising application and economical value of considerable commercial significance and that it could be considered as a potential bio-preservative in the development of control strategies.

## Data Availability Statement

The raw data supporting the conclusion of this article will be made available by the authors, without undue reservation, to any qualified researcher.

## Author Contributions

QW, YD, JZ, and MC conceived the project and designed the experiments. YH, MC, SZ, XW, YZ, JB, and SM performed the experiments. QW and JZ supervised the project. YH, MC, and SZ analyzed the data and wrote the manuscript. YH complemented the writing.

## Conflict of Interest

The authors declare that the research was conducted in the absence of any commercial or financial relationships that could be construed as a potential conflict of interest.

## Footnotes

1 http://www.ncbi.nlm.nih.gov
