# Supplementary material for: Identification of the Potential Biological Preservative Tetramycin A-Producing Strain and Enhancing Its Production
Source: Front Microbiol. 2020 Jan 14;10:2925. doi: 10.3389/fmicb.2019.02925 (PMC6971103; doi:10.3389/fmicb.2019.02925)
Supplement: Supplementary file 1 [file Image_1.pdf]

Supplementary Material

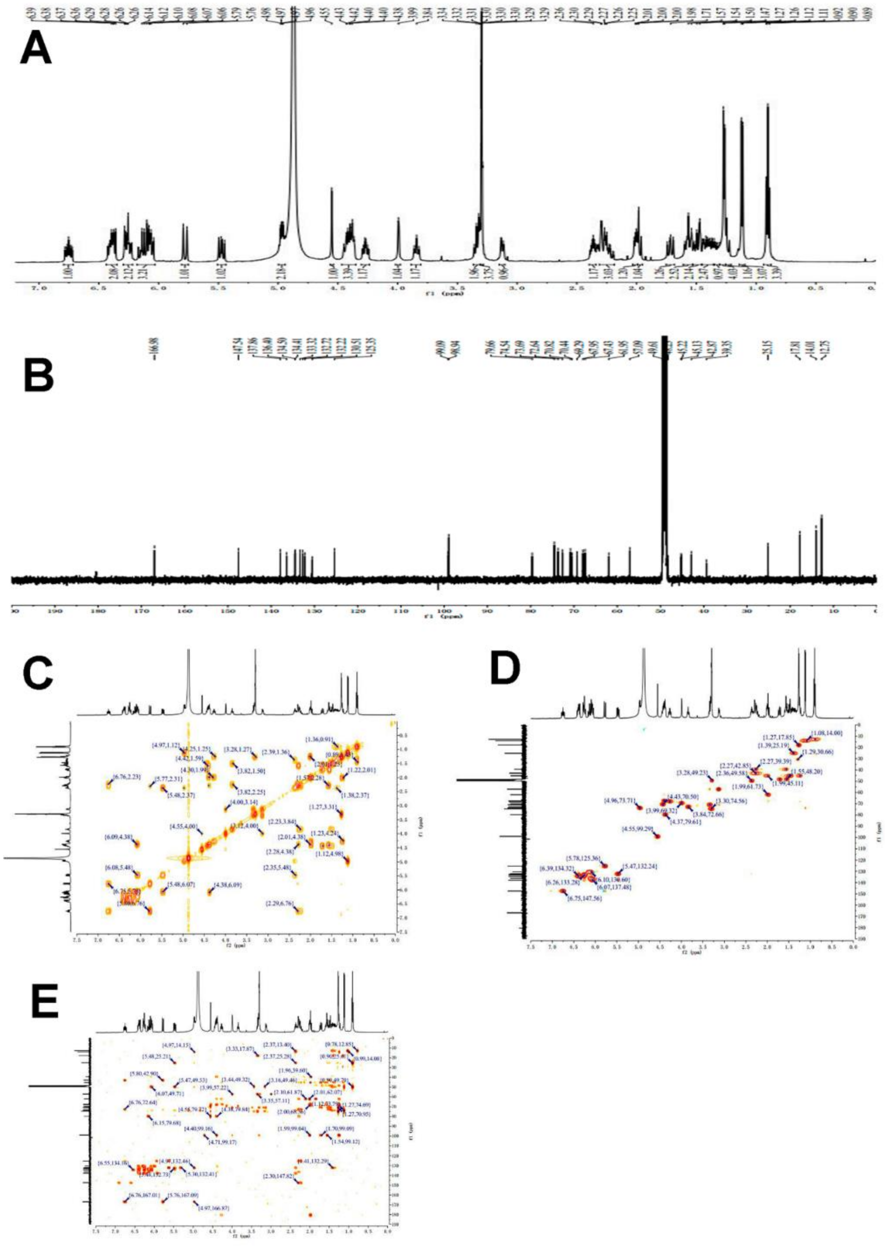

Supplementary Figure 1. NMR spectra of TMA, including ^{1}H-NMR spectrum (A), ^{13}C -NMR spectrum (B), CQSY NMR spectrum (C), HSQC NMR spectrum (D), and HMBC NMR spectrum (E).
